# BY-SLAM: Dynamic Visual SLAM System Based on BEBLID and Semantic Information Extraction

**DOI:** 10.3390/s24144693

**Published:** 2024-07-19

**Authors:** Daixian Zhu, Peixuan Liu, Qiang Qiu, Jiaxin Wei, Ruolin Gong

**Affiliations:** College of Communication and Information Engineering, Xi’an University of Science and Technology, Xi’an 710054, China; 21207223115@stu.xust.edu.cn (Q.Q.); 22207223055@stu.xust.edu.cn (J.W.); 22207223096@stu.xust.edu.cn (R.G.)

**Keywords:** visual SLAM, BEBLID, YOLOv8s, FasterNet, epipolar constraint, clustering

## Abstract

SLAM is a critical technology for enabling autonomous navigation and positioning in unmanned vehicles. Traditional visual simultaneous localization and mapping algorithms are built upon the assumption of a static scene, overlooking the impact of dynamic targets within real-world environments. Interference from dynamic targets can significantly degrade the system’s localization accuracy or even lead to tracking failure. To address these issues, we propose a dynamic visual SLAM system named BY-SLAM, which is based on BEBLID and semantic information extraction. Initially, the BEBLID descriptor is introduced to describe Oriented FAST feature points, enhancing both feature point matching accuracy and speed. Subsequently, FasterNet replaces the backbone network of YOLOv8s to expedite semantic information extraction. By using the results of DBSCAN clustering object detection, a more refined semantic mask is obtained. Finally, by leveraging the semantic mask and epipolar constraints, dynamic feature points are discerned and eliminated, allowing for the utilization of only static feature points for pose estimation and the construction of a dense 3D map that excludes dynamic targets. Experimental evaluations are conducted on both the TUM RGB-D dataset and real-world scenarios and demonstrate the effectiveness of the proposed algorithm at filtering out dynamic targets within the scenes. On average, the localization accuracy for the TUM RGB-D dataset improves by 95.53% compared to ORB-SLAM3. Comparative analyses against classical dynamic SLAM systems further corroborate the improvement in localization accuracy, map readability, and robustness achieved by BY-SLAM.

## 1. Introduction

Simultaneous localization and mapping (SLAM) technology, as one of the key technologies in the field of robotics [1], has been widely used in AR, VR, and robotics. SLAM aims to enable robots to simultaneously achieve autonomous localization and environment map construction in unknown environments with only the sensors they carry. Depending on the sensors carried, SLAM technology is divided into two categories: visual SLAM (VSLAM) [2] and laser SLAM [3]. Visual SLAM is smaller and less expensive than laser SLAM, and it can extract semantic information from collected images; thus, it has become a hotspot for many scholars. After decades of development, many mature visual SLAM systems have emerged, such as MonoSLAM [4], PTAM [5], LSD-SLAM [6], and the Oriented-FAST-and-Rotated-BRIEF-based SLAM (ORB-SLAM) series [7,8,9]. In 2020, Campos et al. proposed ORB-SLAM3 [9], which is recognized as one of the most representative and accurate visual SLAM systems and has excellent robustness. ORB-SLAM3 integrates an inertial measurement unit (IMU) based on ORB-SLAM2 [8] and introduces multi-map reuse technology. However, traditional VSLAM algorithms still have some limitations. For instance, fewer feature points are extracted in weakly textured regions, and a large number of false matches occur in the presence of repetitive textures. Most importantly, traditional VSLAM relies on static scene assumptions and ignores the effects of dynamic targets in real-world environments [10]. However, dynamic targets are a constant in actual situations, which can lead to reduced localization accuracy or even tracking failure.

To solve this problem, numerous scholars have proposed various methods to optimize the traditional VSLAM system. Some researchers have used RANSAC [11] and geometric methods [12,13,14,15] to cull out dynamic feature points in the scene. However, these algorithms require a deep understanding of the scene, and when objects move slowly or dynamic targets occupy most of the image, it is difficult to obtain a good initial camera pose, and it is easy to mistakenly reject static points, which leads to the failure of geometric methods. As deep learning and computer vision technologies continue to advance, some scholars [16,17,18,19] advocate combining semantic segmentation with VSLAM to eliminate the dynamic targets in the scene through a priori semantic information. However, this method can only deal with predefined categories of dynamic objects, which may lead to misclassification: for example, recognizing a stationary person or a parked car as a dynamic target. In recent years, geometric and deep learning fusion methods [20,21,22,23,24,25,26,27,28] have gradually become a hotspot in VSLAM research; they distinguish dynamic and static targets in a scene by combining semantic segmentation with multi-view geometry, motion consistency detection, and epipolar constraints. Although semantic segmentation can provide better pixel-level masks, mask edges are often not segmented accurately enough and have poor real-time performance. Object detection can provide the bounding box of the whole object, but the bounding box often contains the background region, and the feature points in the background region are all valid feature points without the need for geometric constraints. Applying geometric constraints directly to the bounding box region will increase the algorithm’s time consumption. Additionally, these algorithms do not consider the issue of decreased pose estimation accuracy and tracking failures that arise when the total number of feature points decreases after eliminating the dynamic feature points.

To address the above problems, we propose BY-SLAM: a dynamic visual SLAM system based on a boosted efficient binary local image descriptor (BEBLID) [29] and YOLOv8s for semantic information extraction. This system aims to ensure the real-time performance of the SLAM algorithm while effectively reducing the impact of dynamic objects in the scene on the SLAM algorithm. Additionally, it addresses the issue of the significant reduction in the total number of feature points caused by the elimination of dynamic feature points, which leads to a decrease in the accuracy of the SLAM system’s pose estimation and tracking failures.

The primary innovative aspect of this paper is outlined as follows:

(1) The lightweight FasterNet [30] is employed to substitute the backbone of YOLOv8s in order to speed up algorithmic inference. Then, YOLOv8s-FasterNet is integrated into the ORB-SLAM3 system, enabling real-time processing of bounding boxes for potential dynamic objects.
(2) The issue of excessively large object detection bounding boxes leads not only to dynamic targets being included within the bounding boxes but also a significant amount of background. To address this issue, we propose utilizing density-based spatial clustering of applications with noise (DBSCAN) density clustering to extract semantic information within the detection boxes and generate semantic masks. Subsequently, dynamic feature points are filtered out by integrating epipolar constraints.
(3) To address the issue of tracking and localization failure resulting from a notable decrease in feature points following the elimination of dynamic feature points, we propose utilizing the BEBLID instead of the rotated binary robust independent elementary features (rBRIEF) descriptor to improve matching accuracy and efficiency.
(4) Addressing the limitations of sparse mapping and the issue of excessive ghosting in ORB-SLAM3, this paper introduces a dense 3D mapping thread into the ORB-SLAM3 framework. Through strategies such as dynamic target ghost elimination, statistical filtering, and voxel downsampling, a dense 3D map with low noise is successfully constructed.

This paper’s remaining structure is as follows: Work on dynamic SLAM is described in Section 2. Section 3 presents the components of the BY-SLAM system. Comparative analyses and experimental verification are conducted in Section 4. Finally, this article is summarized in Section 5.

## 2. Related Work

### 2.1. Geometric Methods

Geometric-based SLAM primarily employs motion consistency checks, multi-view geometry, or epipolar constraints to identify and eliminate feature points that do not meet geometric requirements as dynamic. Wang et al. [12] used geometric constraints and clustered depth images to identify and filter matching feature points between neighboring frames and to remove dynamic targets. Dai et al. [13] proposed a method using Delaunay triangulation [31] to determine static feature points. All the map points are connected by Delaunay triangulation, and the connecting lines indicate the correlation. After removing the connecting lines between the irrelevant points, the largest remaining group is the static map points. Lu et al. [14] performed background restoration on the image by embedding it into the BundleFusion framework and then employed geometric residuals to eliminate dynamic elements, followed by localization and tracking of the restored background. Song et al. [15] suggested using a combination of geometric consistency, epipolar constraints, and DBSCAN clustering to remove dynamic targets.

### 2.2. Deep Learning Methods

Scholars have extended methods like object detection, semantic segmentation, and instance segmentation to the realm of dynamic SLAM as a result of the ongoing advancements in deep learning. Zhong et al. [16] proposed a Detect-SLAM system based on SSD [32] that selects only key frames for detection, updates the movement probabilities according to the detection results, and rejects dynamic targets through movement probability propagation while constructing a semantic map. Henein et al. [17] presented a dynamic SLAM algorithm without model priors that uses semantic segmentation to estimate the motion of rigid bodies in the scene rather than requiring the estimation of the object’s pose or any other a priori information of the 3D model. Liu et al. [18] introduced RDS-SLAM, which employs SegNet [33] to obtain semantic information, and proposed a key frame selection strategy. Meanwhile, movement probability is utilized to convey the semantic information to boost the algorithm’s real-time efficiency. Zhang et al. [19] proposed a dynamic SLAM system based on the combination of IMU and segmentation methods that uses IMU data to calculate the movement probability of the feature points and updates the movement probabilities according to the segmentation results, thereby eliminating feature points related to dynamic objects.

### 2.3. Geometric and Deep Learning Fusion Methods

In recent years, geometric and deep learning fusion methods have gradually become a hotspot in VSLAM research. DynaSLAM was proposed by Bescos et al. [20]. It combines multi-view geometry with a Mask R-CNN [34] semantic segmentation network to remove dynamic objects and adds a background repair function; however, the algorithm cannot meet real-time requirements due to Mask R-CNN semantic segmentation’s high processing time. Yu et al. [21] proposed DS-SLAM, which uses SegNet [33] combined with motion consistency tests to remove dynamic targets in the scene and generate dense semantic octree maps. However, its capacity to identify targets is limited to 20, and it exclusively categorizes people as a priori moving categories, resulting in poor robustness. Xiao et al. [22] proposed a leakage compensation algorithm to improve the detection recall of the SSD [32] and using selective tracking algorithms in the tracking thread to handle dynamic targets. Bescos et al. [23] introduced DynaSLAM II, which is a method that utilizes instance semantic segmentation and ORB features for the purpose of tracking dynamic targets. They proposed a solution based on bundle adjustment (BA) [35] that tightly optimizes the scene structure, camera pose, and object trajectory in a local time window and also optimizes the bounding box of the object using a decoupled formulation. Fan et al. [24] used YOLAC [36] for object instance segmentation and combined depth information to eliminate dynamic features. They suggested a technique to remove ghosting created by moving targets from point cloud maps, thereby eliminating the impact of dynamic targets in the created maps. Liu et al. proposed Dynamic-VINS [25], which estimates the camera pose using a fusion of RGB-D camera and IMU data and removes dynamic feature points from the scene by combining YOLOv3 [37] and depth information. But it does not construct a map with the dynamic objects removed. Ye et al. proposed DeFlowSLAM [26], which uses a dual-flow representation to decompose the optical flow in a scene into a static flow field and a dynamic flow field to estimate the camera pose. Zhang et al. [27] proposed a method to detect dynamic objects in the scene using Ghostnet [38] and YOLOv5 improved by the coordinate attention mechanism; their method eliminates all the feature points in the object detection box. If the dynamic targets occupy most of the object detection box, this method will lead to tracking failure. Lin et al. [28] used point-line feature fusion for feature extraction; they utilized object detection to extract a priori dynamic targets and combined global optical flow and the epipolar constraints to further eliminate the dynamic feature points and feature lines.

At present, methods combining geometry and deep learning have achieved good performance at rejecting dynamic feature points in dynamic scenes. However, SLAM systems in dynamic scenes still face issues such as tracking and localization failures due to the massive rejection of feature points on dynamic targets or the inability to meet real-time requirements. Therefore, we propose BY-SLAM: a SLAM algorithm designed for dynamic environments. It utilizes BEBLID descriptors, YOLOv8s-FasterNet, DBSCAN density clustering, and epipolar constraints to remove dynamic feature points and construct dense 3D maps that eliminate dynamic targets.

## 3. Methods

### 3.1. System Overview

**Figure 1:**
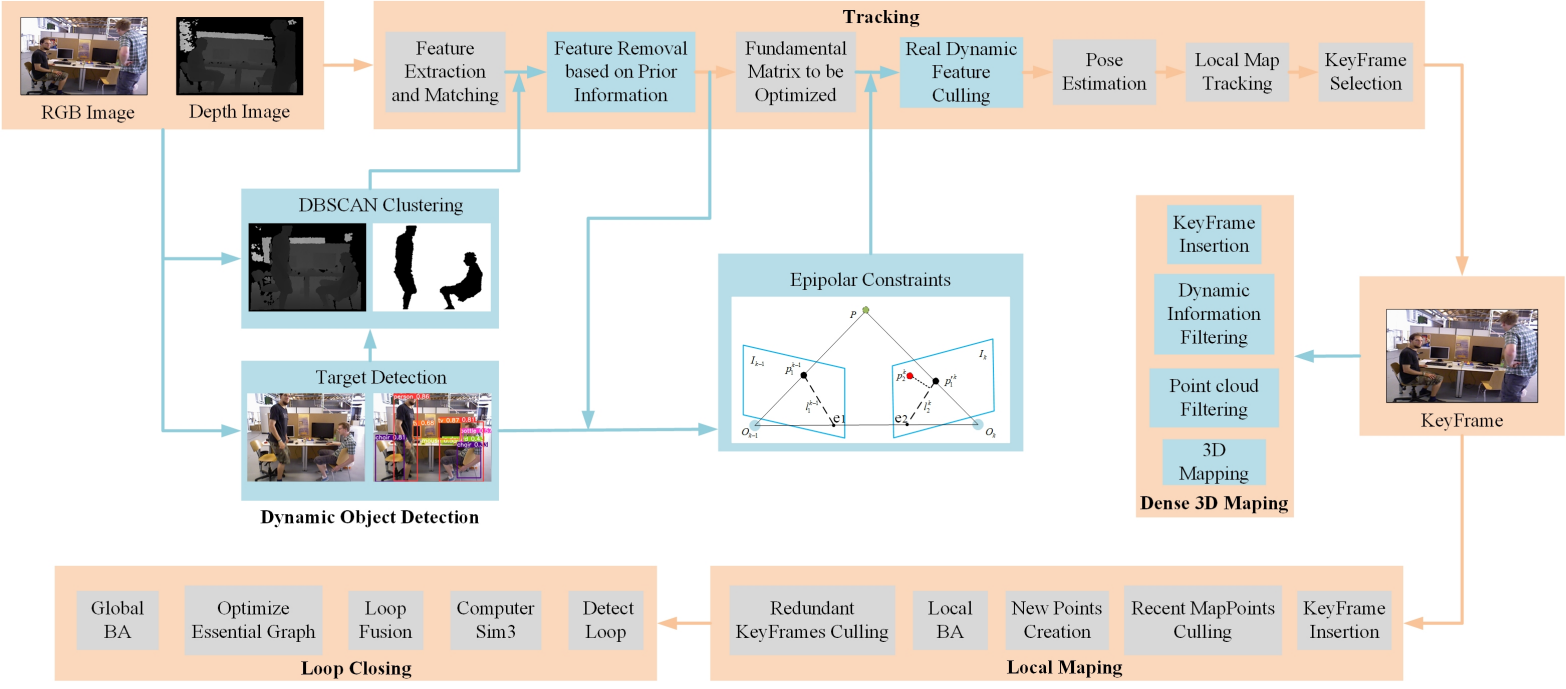
Framework of BY-SLAM. Two new threads have been added to the ORB-SLAM3 framework: dynamic object detection and dense 3D mapping. The ORB-SLAM3 framework is shown in gray; the new features added to BY-SLAM are shown in blue.

BY-SLAM uses ORB-SLAM3 [9] as the foundational framework for algorithmic improvement. The ORB-SLAM3 system is recognized as one of the most classical visual SLAM systems, and it primarily comprises three threads: tracking, local mapping, and loop closing. We add new parallel threads for dynamic object detection, dense mapping, and dynamic feature points elimination to the ORB-SLAM3 system. Additionally, the original rBRIEF descriptor is replaced with BEBLID. The updated system framework is depicted in Figure 1.

Initially, Oriented FAST feature points extracted from the current image are described using the BEBLID descriptor. Meanwhile, the lightweight YOLOv8s-FasterNet is employed to identify a priori dynamic targets in RGB image sequences. The detected frames are then mapped to corresponding depth image sequences, and the semantic mask of the images is extracted using the DBSCAN density clustering algorithm. The image is separated into static and potentially dynamic parts based on its semantic mask combined with predefined prior information. Feature points located outside the semantic mask are directly classified as static feature points for subsequent tasks. Subsequently, those within the semantic mask undergo further constraint by epipolar geometry to preserve more static feature points. Finally, static feature points are employed for tracking and pose estimation, and the updated poses and keyframes with dynamic pixels removed are passed into the dense mapping thread to reconstruct the global static dense 3D map.

### 3.2. BEBLID Descriptor

Image matching is an extremely important component of visual SLAM, and its accuracy relies on the feature point extraction method and descriptor selection. The ORB feature extraction scheme, namely, Oriented FAST feature points and rBRIEF descriptors, is employed in the ORB-SLAM3 system for extracting and describing feature points; rBRIEF, an adaptation of BRIEF, aims to enhance the robustness of descriptors against image rotation, thereby achieving rotational invariance to some extent. However, in comparison to the original BRIEF algorithm, rBRIEF exhibits certain drawbacks in terms of descriptor distinguishability, resulting in higher similarity between descriptors, which may lead to erroneous matches. Therefore, BY-SLAM replaces the rBRIEF descriptor with BEBLID, which offers greater robustness and higher matching accuracy. The BEBLID descriptor provides more precise and efficient feature representation, with significantly improved distinguishability, thereby enhancing feature matching accuracy. Moreover, it addresses the issue of insufficient feature points after dynamic feature removal, consequently further improving the tracking and localization accuracy.

BEBLID [29] is a binary descriptor proposed by Suárez et al. that is based on the BELID [39] algorithm. BEBLID introduces the AdaBoost algorithm and an improved weak learner training scheme to achieve more precise local descriptions. It was trained on the Brown dataset [40], wherein the training samples are pairs of labeled image patches:

(1) $${\left\{(x_{i},y_{i},l_{i})\right\}}_{i=1}^{N}$$

where $x_{i},y_{i}\in X$ represent image block pairs, $l_{i}\in \left\{-1,1\right\}$ represent the labels in the training samples, $l_{i}=1$ indicates that the two image blocks have the same feature structure, and $l_{i}=-1$ indicates that they are different.

(2) $$L_{\mathrm{BEBLID}}=\sum_{i=1}^{N}\exp\left(-\gamma l_{i}\sum_{k=1}^{K}h_{k}\left(x_{i}\right)h_{k}\left(y_{i}\right)\right)$$

where γ is the weak learner weight, and $h_{k}\left(x\right)\equiv h_{k}\left(x;f,T\right)$ is the *k*-th weak learner defined using the feature extraction function f(x) and threshold *T*. Define the weak learner function as follows:

(3) $$h\left(x,f,T\right)=\left\{\begin{array}{cc} +1 & \mathrm{if}\;f(x)\leq T\\ -1 & \mathrm{if}\;f(x)>T \end{array}\right.$$

The feature extraction function $f(x;p_{1},p_{2},s)$ of BEBLID calculates the difference between the average gray values of the pixels in the two square regions $R(p_{1},s)$ and $R(p_{2},s)$. The feature extraction function is defined as follows:

(4) $$f\left(x;p_{1},p_{2},s\right)=\frac{1}{S^{2}}\left[\sum_{q\in R(p_{1},s)}I\left(q\right)-\sum_{r\in R(p_{2},s)}I\left(r\right)\right]$$

where I(t) represents the gray value at *t*, and R(p,s) represents the sum of gray values in the rectangular region centered at *p* with a side length of *s*. The description symbol function is defined as follows:

(5) $$D\left(x\right)=A^{\frac{1}{2}}h\left(x\right)={\left[\sqrt{\alpha_{1}}h_{1}\left(x\right)\cdots \sqrt{\alpha_{k}}h_{k}\left(x\right)\right]}^{T}$$

where $A=\mathrm{diag}(\alpha_{1},\ldots ,\alpha_{k})$; $\alpha_{i}$ is the weight assigned to the *i*-th weak learner by the AdaBoost algorithm. When all weak learners share the same weight, a binary descriptor is generated.

**Figure 2:**
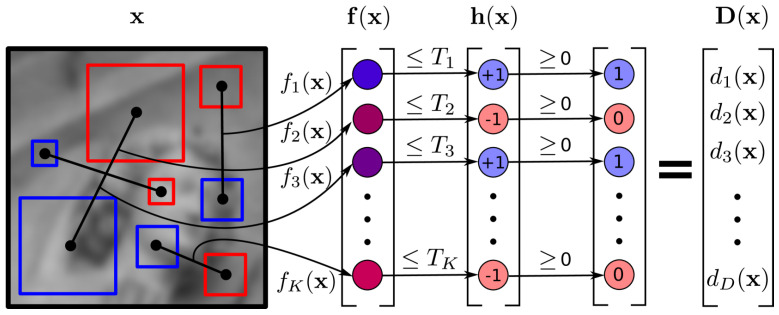
BEBLID descriptor extraction flow.

Figure 2 illustrates the extraction process for BEBLID descriptors. $R(p_{1},s)$ and $R(p_{2},s)$ are the blue and red regions in the figure, respectively. The difference between the average gray levels of these two regions is calculated by the feature extraction function. Then, the parameters and weights in AdaBoost are adjusted to optimize the loss function, thereby differentially selecting a set of features. Eventually, the optimal BEBLID binary descriptor is obtained by continuously iterating the pixel positions and sizes of the descriptor.

### 3.3. YOLOv8s-FasterNet Object Detection Network

Deep-learning-based dynamic SLAM algorithms usually use object detection or semantic segmentation to predict a priori dynamic targets. However, object detection runs faster than semantic segmentation algorithms and has less stringent computational power requirements. To ensure the real-time performance of the SLAM system, we use object detection to predict a priori dynamic targets. The YOLO series [41] is a single-stage regression-based algorithm based on deep learning; it gains faster operation while slightly losing accuracy compared to two-stage classification algorithms. The network structure of YOLOv8 is primarily composed of backbone, neck, and head modules. The head network adopts the decoupled-head structure, which separates the classification and detection heads, and replaces the anchor-base with the anchor-free detection head. The task-aligned assigner positive and negative sample allocation strategy was used for the loss part, and distribution focal loss (DFL) was introduced. The mosaic enhancement technique was used during training, but it was disabled in the last 10 epochs to mitigate potential effects throughout the training process. YOLOv8 contains five network models: n, s, m, l, and x. The depth of the network models and the width of the feature maps gradually increase, and the detection accuracy gradually increases. YOLOv8s has a slightly higher number of parameters and runtime speed compared to YOLOv8n, but the accuracy of detection has been significantly enhanced. Therefore, we select the YOLOv8s model for improvement.

**Figure 3:**
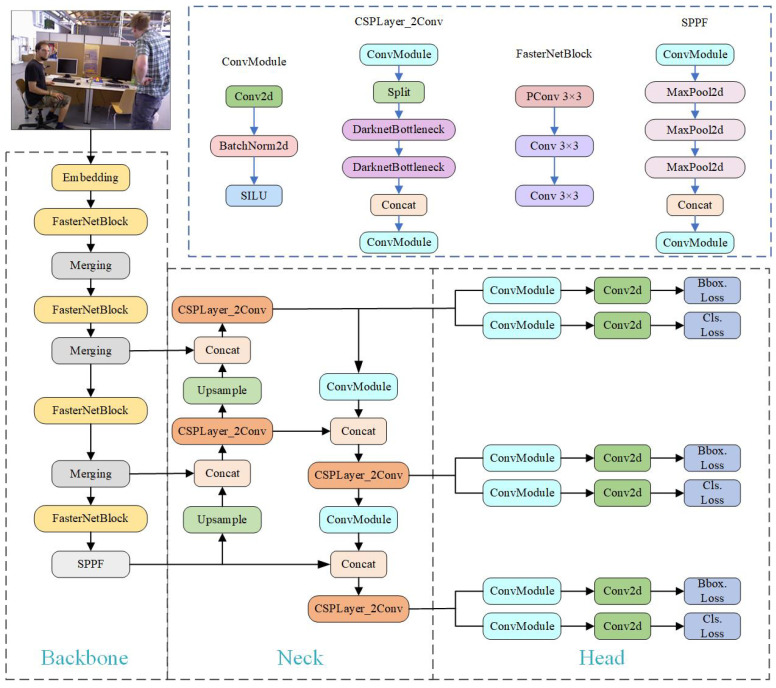
Framework of the proposed YOLOv8s-FasterNet.

In the BY-SLAM system proposed in this paper, adding dynamic target detection threads increases the elapsed time. To ensure the real-time performance of the SLAM system, a lightweight YOLOv8s model is required. Table 1 demonstrates the performance comparison of the lightweight FasterNet [30], MobileViT [42], MobileNetV2 [43], and PVT [44] on different devices. The data in the table, cited from ref. [30], show the throughput and latency performance of these network models on GPU (NVIDIA 2080Ti, Santa Clara, CA, USA), CPU (Intel i9-9900X, Santa Clara, CA, USA), and ARM (ARM Cortex-A72, Cambridge, UK) devices as well as their parameter counts and accuracy. It can be seen that FasterNet runs optimally on different devices. Therefore, we chose to use FasterNet to replace the backbone of YOLOv8s, and the YOLOv8s-FasterNet structure is shown in Figure 3.

**Figure 4:**
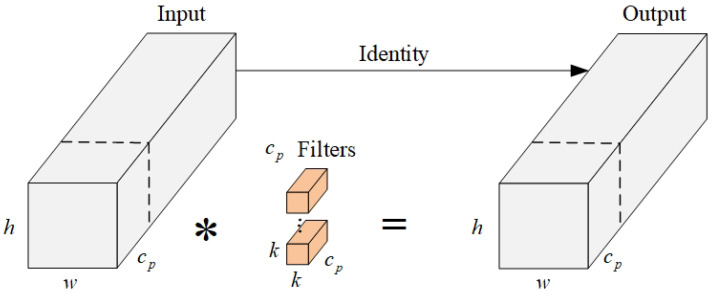
Principle of PConv.

Partial convolution (PConv) is a new partial convolution method proposed by Chen et al. [30]. This method speeds up algorithm operation by reducing memory access requirements and redundant computations and is able to retain more spatial features. The principle of PConv is shown in Figure 4. Based on the idea of PConv, the authors further propose FasterNet: a lightweight backbone network that runs much faster on various devices without affecting algorithm accuracy.

PConv selectively convolves certain input channels, leaving the rest unconvolved. The FLOPs and memory access requirements of PConv are shown in Equations (6) and (7):

(6) $$h\times w\times k^{2}\times c_{p}^{2}$$

(7) $$h\times w\times 2c_{p}+k^{2}\times c_{p}^{2}\approx h\times w\times 2c_{p}$$

where *h* and *w* are the width and height of the feature map, *k* is the size of the convolution kernel, $c_{p}$ is the number of partial convolution channels, and *c* is the number of all convolution channels. In general, $r=\frac{c_{p}}{c}=\frac{1}{4}$; thus, the FLOPs and memory access requirements of PConv are 1/16 and 1/4 those of a regular Conv, respectively. The remaining $\left(c-c_{p}\right)$ channels do not undergo convolution and do not require memory access.

### 3.4. Dynamic Feature Point Elimination

Semantic segmentation can directly produce masks for targets, but object detection uses regular rectangular sections called bounding boxes to represent targets. In addition to dynamic targets, these bounding boxes frequently include a large number of static targets. Based on how they move, things in the scene can be divided into two categories: dynamic targets—for example, pedestrians, pets, and vehicles—and static targets—for example, tables, chairs, and unmoving people. Directly removing every feature point within the bounding box may erroneously delete static feature points, leading to a decrease in pose estimation accuracy and even tracking failure. BY-SLAM suggests a new thresholding model based on DBSCAN clustering and epipolar constraints to detect dynamic targets in the bounding box, with the goal of retaining as many static feature points as possible.

#### 3.4.1. DBSCAN Clustering

RGB-D cameras can provide depth information in the environment. Typically, the surface depth values of dynamic targets are very different from those of the background depth. This difference makes it easier to separate objects with complete contours by clustering the depth images. Therefore, this paper introduces the DBSCAN algorithm to quantitatively cluster the depth images to distinguishing the foreground from the background within the bounding box. For the key parameters of this algorithm, the neighborhood radius (eps) and the threshold of the number of samples (MinPts) are determined adaptively. After clustering is completed, the set of points in the cluster $C=\{C_{1},C_{2},\cdots ,C_{k}\}$ with the smallest mean depth and the highest number of points is used as the foreground of the detection frame. The fundamental matrix is then computed using every feature point except those within the mask, followed by estimation of the pose.

**Figure 5:**
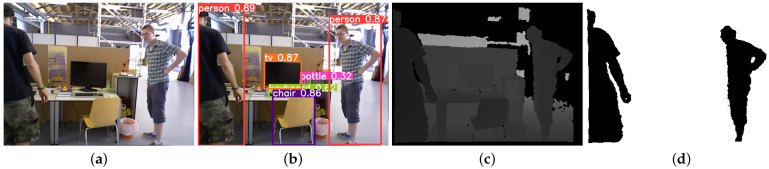
DBSCAN clustering experiment. (**a**) RGB image of the 90th frame of the fr3_walking_xyz sequence; (**b**) object detection results; (**c**) depth image of the 90th frame of the fr3_walking_xyz sequence; (**d**) DBSCAN clustering results.

The result of DBSCAN clustering is shown in Figure 5, which illustrates that the outline of the person is more complete and the foreground and background are well segmented. Therefore, the use of DBSCAN clustering can obtain a finer semantic mask in the scene, significantly reducing the adverse effects of an oversized object detection frame and improving the accuracy of subsequent pose estimation.

#### 3.4.2. Epipolar Constraints

**Figure 6:**
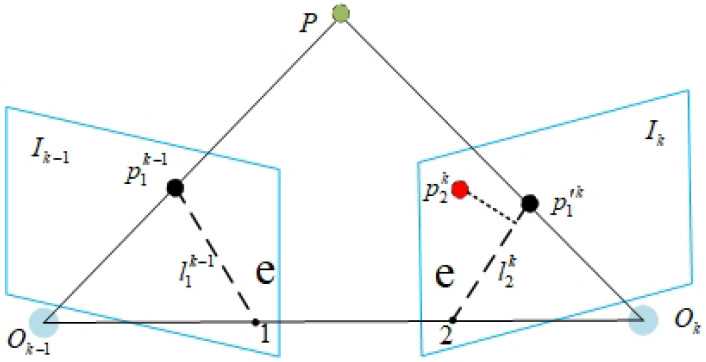
Epipolar constraint.

YOLOv8s only recognizes object categories and cannot determine the state of objects. Therefore, epipolar constraints must be used to categorize the target’s current state, as illustrated in Figure 6. Feature points belonging to dynamic objects cannot satisfy the epipolar constraints. Thus, this paper calculates the separation between feature sites and their corresponding epipolar lines and considering feature points whose distance exceeds a predetermined threshold as outliers.

After the RGB-D camera acquires the RGB and depth images, a relatively reliable fundamental matrix $F_{i}$ between frames is initially computed using feature points located outside the semantic mask. Then, the matrix $F_{i}$ is used to check whether the feature points in the experimental area satisfy the epipolar constraint. Assuming that point *P* in the world coordinate system projects to point $p_{1}^{k-1}$ on the camera plane $I_{k-1}$ at the optical center $O_{k-1}$ and projects to point $p_{1}^{\prime k}$ on the camera plane $I_{k}$ at the optical center $O_{k}$, the matching point of $p_{1}^{k-1}$ is $p_{2}^{k}$. The 3D coordinates of point *P* and the projection point coordinates are shown in Equations (8) and (9):

(8) $$P={\left[X,Y,Z\right]}^{⊤}$$

(9) $$\left\{\begin{array}{c} p_{1}^{k-1}={\left[u_{1},v_{1},1\right]}^{⊤}\\ p_{2}^{k}={\left[u_{2},v_{2},1\right]}^{⊤} \end{array}\right.$$

where $l_{2}^{k}$ is the intersection line of the epipolar plane $O_{k-1}O_{k}P$ formed by points $p_{1}^{k-1}$, $p_{1}^{\prime k}$, and *P* with plane $I_{k}$. Based on the transformation relationship between world coordinates and pixel coordinates and the camera’s intrinsic matrix *K*, then:

(10) $$\left\{\begin{array}{c} p_{1}^{k-1}=KP\\ p_{2}^{k}=K\left(RP+t\right) \end{array}\right.$$

By combining Equations (9) and (10), we obtain:

(11) $${\left(p_{2}^{k}\right)}^{⊤}K^{-T}\hat{t}RK^{-1}p_{1}^{k-1}=0$$

where $\hat{t}$ is the skew-symmetric matrix of *t*. Equation (11) indicates that the static point *P* in the world coordinate system and its projections $p_{1}^{k-1}$ and $p_{1}^{\prime k}$ on the camera planes $I_{k-1}$ and $I_{k}$, respectively, must satisfy the epipolar line constraint. Combined with the fundamental matrix $F_{i}$, then:

(12) $$F_{i}=K^{-T}\hat{t}RK^{-1}$$

*A* and *B* are the line parameters of $l_{2}^{k}$ obtained based on $p_{1}^{\prime k}$ and $F_{i}$, and *d* is the distance from $p_{2}^{k}$ to the epipolar line $l_{2}^{k}$, as shown in Equation (13):

(13) $$d=\frac{{\left(p_{2}^{k}\right)}^{⊤}F_{i}p_{1}^{k-1}}{\sqrt{A^{2}+B^{2}}}$$

Then *d* can be used to verify dynamic key points. For static points, $d\leq d_{\mathrm{th}}$, while for dynamic feature points, $d>d_{\mathrm{th}}$, where $d_{\mathrm{th}}$ is the epipolar threshold parameter. After using the epipolar constraint to remove all dynamic feature points, the initial fundamental matrix $F_{i}$ is updated using only static feature points, obtaining the final fundamental matrix *F* for subsequent pose estimation tasks.

### 3.5. Dense 3D Map Construction

Once the current frame’s pose information has been obtained, first, the dynamic pixels in the keyframes are filtered out. Second, the filtered keyframes and camera pose information are passed into the dense map building thread to incrementally construct the point cloud map, which is constituted by points with colors and 3D coordinates. Finally, the noise is further filtered out using statistical filtering and point cloud downsampling techniques. In turn, a global static dense 3D map is obtained. Compared with the sparse point cloud map, which is the only type of map that can be constructed by ORB-SLAM3, the dense 3D map created by BY-SLAM that excludes dynamic targets can more effectively represent indoor scene information. This is convenient for practical applications such as robot navigation and obstacle avoidance.

## 4. Experiments

To ascertain the effectiveness of BY-SLAM, we tested it using the indoor dynamic sequences from the TUM RGB-D public dataset [45] and a self-made real-world scene dataset. The configuration of the simulation experiment platform is as follows: Ubuntu 18.04, Intel i9-12900K CPU @ 3.90 GHz, RTX 3080Ti GPU, and 32 GB of RAM.

### 4.1. Image Matching Experiment

**Figure 7:**
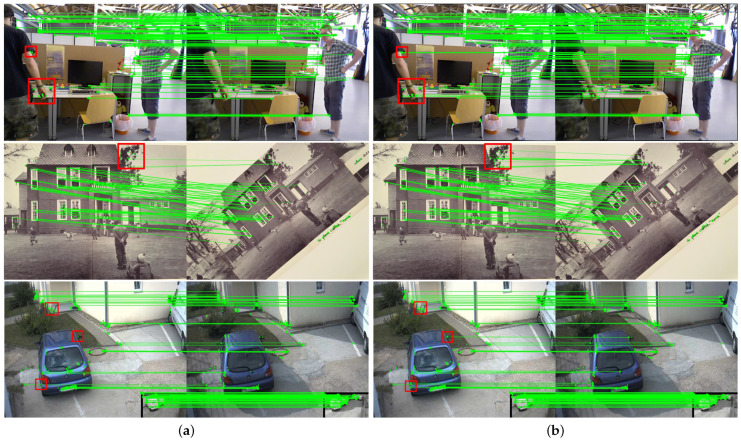
Feature matching results. The green lines represent the feature correspondences between matching points. Different scenes are represented from top to bottom and are consecutive frames of the fr3_walking_xyz dynamic sequence, v_home, and i_parking (1–3). (**a**) ORB algorithm results; (**b**) results of our algorithm.

In this paper, we select any consecutive frames from the fr3_walking_xyz dynamic sequence of the TUM RGB-D dataset and a total of three sets of images from the v_home and i_parking sequences of the HPatches dataset [46], and we carry out image matching experiments. The dataset is based on several small-scale datasets that have been previously proposed and extended in the field of image matching, and it mainly focuses on the view angle changes of a flat scene and the light changes of a general scene. It contains two subsets corresponding to sets of images with different light changes and sets of images with different view angle changes, respectively, in which v denotes the view angle change and i denotes the light change. During the experiment, 500 feature points are extracted using Oriented FAST, and the feature points are described using rBRIEF and BEBLID descriptors, respectively. The feature points are matched using a brute-force matcher with the Hamming distance. The number of inliers in the matches is calculated using the homography matrix, and the matching rate is the percentage of inliers to the overall matches. The matching outcomes are depicted in Figure 7. The red square region distinctly indicates that the algorithm employing BEBLID descriptors is capable of matching more pairs of feature points successfully. This is due to the fact that rBRIEF has rotational invariance, which sacrifices part of the distinguishability, while BEBLID is a descriptor based on deep learning and is able to better describe the local features, resulting in higher matching accuracy for the same threshold.

Table 2 quantifies the feature matching results and indicates that for the same threshold, the average matching rate of this paper’s algorithm is improved by 13.8% relative to the ORB algorithm, and the average feature matching computational efficiency is also improved by 26.37%, which enhances the matching accuracy and speed of feature point. In summary, compared with the ORB algorithm, the Oriented FAST keypoints proposed in this paper combined with the BEBLID descriptor obtain more inliers, higher matching accuracy, shorter computation time, and stronger adaptability and robustness in complex scenes. This can improve the pose estimation accuracy of the SLAM system.

### 4.2. Lightweight Object Detection Network Validation

To verify the performance of YOLOv8s-FasterNet, the COCO2017 dataset, which contains 80 categories covering common dynamic objects such as people, pets, and vehicles, is used for training. The training hyperparameter settings are shown in Table 3.

After the training is completed, the performance of YOLOv8s-FasterNet is tested against each model of the YOLOv8 series on the CPU, as shown in Table 4. The metric mAP is utilized for assessing the accuracy of predictions, with higher values indicating better performance; the Inference Time per Frame is used to measure the operation efficiency, with smaller values indicating faster rates; Params are employed for evaluating the size of the model, with smaller values indicating lighter models; and FLOPs stands for the number of floating-point operations, with smaller values indicating fewer computations. It is evident from Table 4 that the Params of YOLOv8s-FasterNet decrease by 43.01% compared to YOLOv8s, and the inference speed on the CPU increases by 46.89%, but the object detection accuracy decreases by 6.2%. This is due to the fact that FasterNet, as a lightweight backbone network, falls short in detection performance. Notably, compared to the YOLOv8n network, YOLOv8s-FasterNet improves the detection accuracy by 12.9% with almost the same inference speed. In addition, the inference speed of YOLOv8s-FasterNet on the CPU can reach 29 FPS, which meets the real-time requirement of the SLAM system.

### 4.3. Dynamic Feature Point Elimination Experiments

**Figure 8:**
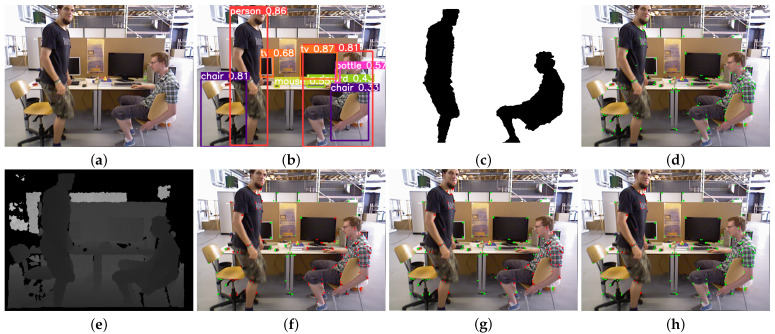
Dynamic feature point elimination experiment for the TUM RGB-D dataset. (**a**) RGB image of the 87th frame of the fr3_walking_static sequence; (**b**) object detection results; (**c**) clustering results of DBSCAN; (**d**) feature extraction results of ORB-SLAM3; (**e**) depth image of the 87th frame of the fr3_walking_static sequence; (**f**) direct rejection of all feature points in the target frame; (**g**) direct elimination of dynamic feature points within the semantic mask; (**h**) the result of eliminating dynamic feature points using our algorithm.

In this section, the dynamic feature filtering algorithm is tested using dynamic sequences from the TUM RGB-D dataset. The dynamic feature point filtering experiment for the TUM RGB-D dataset is shown in Figure 8. The feature points of the original region are marked in green, and the detected dynamic feature points are marked in red.

In Figure 8, the person sitting on the chair remains stationary, while the standing person moves slowly. The algorithm in this paper ultimately recognizes the standing person as a dynamic target and identifies the feature points on their body as dynamic feature points after applying the epipolar constraints. The person sitting on the chair is recognized as a static target, and the static feature points on their body are retained after applying the epipolar constraints. This demonstrates that the dynamic feature point rejection method proposed in this paper can accurately recognize and eliminate the dynamic feature points while retaining as many static feature points as possible for the subsequent pose estimation task.

To further verify the effectiveness of the algorithms proposed in this paper in practical applications, a dataset under real dynamic scenes is self-made. The visual sensor used to collect the environmental information is the Kinect V1 depth camera from Microsoft: the RGB camera has a resolution of 640 × 480 and acquires RGB image information at a video stream rate of 30 frames per second.

**Figure 9:**
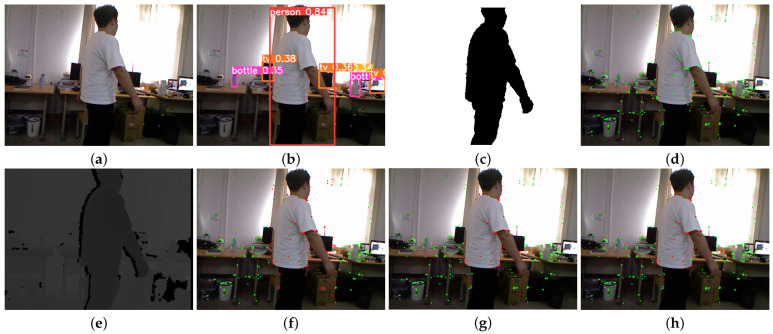
Real-world scene feature point elimination experiment. (**a**) RGB image of frame 163 of the real-world dataset; (**b**) object detection results; (**c**) clustering results of DBSCAN; (**d**) feature extraction results of ORB-SLAM3; (**e**) depth image of frame 163 of the real-world dataset; (**f**) direct rejection of all feature points in the target frame; (**g**) direct rejection of the dynamic feature points within the semantic mask; (**h**) the result of our algorithm for eliminating dynamic feature points.

The effect of our algorithm on dynamic feature point rejection in a real-world scene is shown in Figure 9. The classmates appearing in the laboratory sequence are kept as dynamic, so only the feature points on the person are filtered out, while a large number of static feature points are retained in the background information. This is consistent with the experimental results of the TUM RGB-D dataset.

The experimental findings demonstrate that the dynamic feature point rejection technique introduced in this paper effectively identifies and eliminates genuine dynamic objects within the scene; it retains purely static feature points for pose estimation. This enhancement leads to improved accuracy and robustness in localization for the algorithm.

### 4.4. Trajectory Accuracy Verification Experiment

Absolute trajectory error (ATE) and relative pose error (RPE) are important metrics for determining the performance of an algorithm. ATE represents the absolute value of the distance difference between the estimated camera pose trajectory and the ground truth trajectory and serves as the most intuitive indicator of system accuracy. RPE represents the local accuracy of the estimated pose trajectory over a fixed time interval and includes both translation and rotation errors. In this paper, the EVO [47] evaluation tool is used to assess the system, with root mean square error (RMSE), mean error (Mean), median error (Median), and standard deviation (SD) used for quantitative evaluation of these metrics. Improvements denote the performance improvement of BY-SLAM over the ORB-SLAM3 algorithm and are denoted as α.

(14) $$\alpha =\frac{m-n}{m}\times 100\%$$

where *m* represents the error result of ORB-SLAM3, and *n* represents the error result of BY-SLAM. To avoid the randomness of a single experiment, the experiment was repeated 10 times, and the average value was taken as the final result.

Table 5, Table 6 and Table 7 show the test results of ATE and RPE for ORB-SLAM3 and BY-SLAM. The fr3_walking and fr3_sitting sequences represent the high dynamic and low dynamic categories, respectively. There are four types of camera motions: xyz, static, halfsphere, and rpy. The xyz motion means the camera moves along the spatial 3D orthogonal directions; static means the camera is essentially stationary; halfsphere means the camera moves along a hemispherical trajectory; and rpy means the camera rotates along the roll–pitch–yaw axes. From the tables, it can be seen that BY-SLAM significantly outperforms ORB-SLAM3 in terms of ATE and RPE. The performance improvement is especially noticeable in high dynamic sequences, where RMSE and SD improve localization accuracy by more than 80% compared to ORB-SLAM3. In low dynamic sequences, RMSE and SD improve localization accuracy by more than 14% compared to ORB-SLAM3. This lesser improvement is due to the already good performance of ORB-SLAM3 in low dynamic sequences.

**Figure 10:**
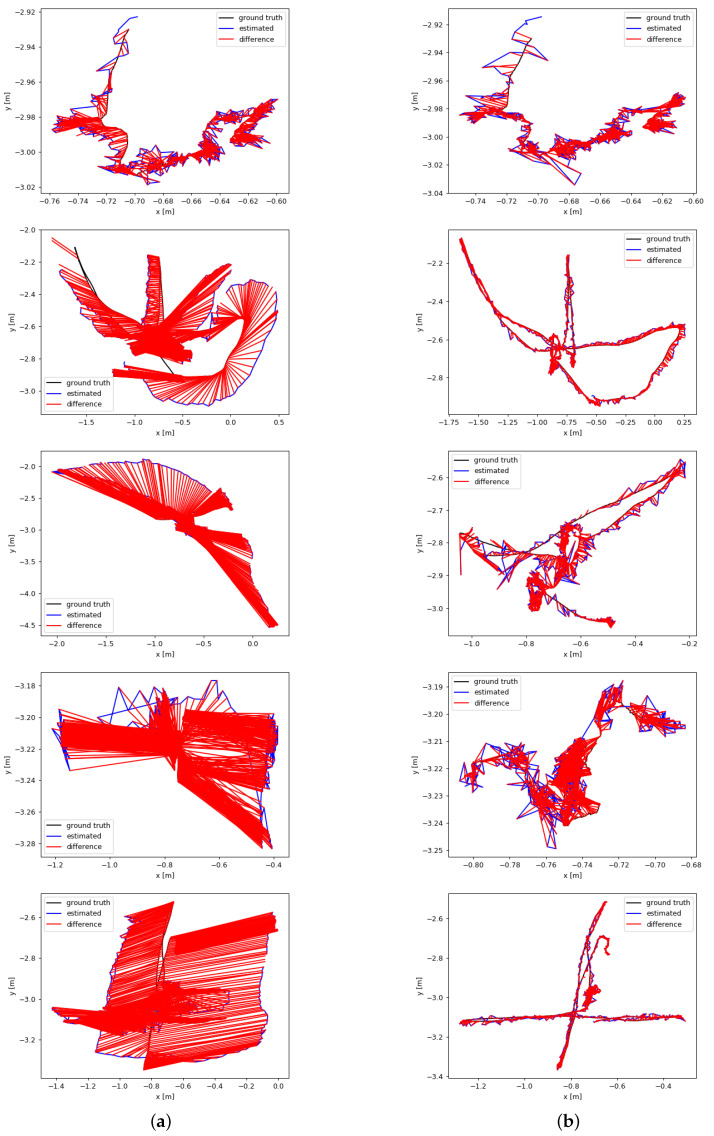
ATE for ORB-SLAM3 and BY-SLAM is evaluated on certain sequences from the TUM RGB-D dataset. In the ATE evaluation, the real camera trajectory is represented by the black line, the estimated camera trajectory is represented by the blue line, and the discrepancy between the two is represented by the red line. Different scenes are represented from top to bottom: (1) fr3_sitting_static, (2) fr3_walking_half, (3) fr3_walking_rpy, (4) fr3_walking_static, and (5) fr3_walking_xyz. (**a**) ORB-SLAM3 algorithm results; (**b**) BY-SLAM algorithm results.

**Figure 11:**
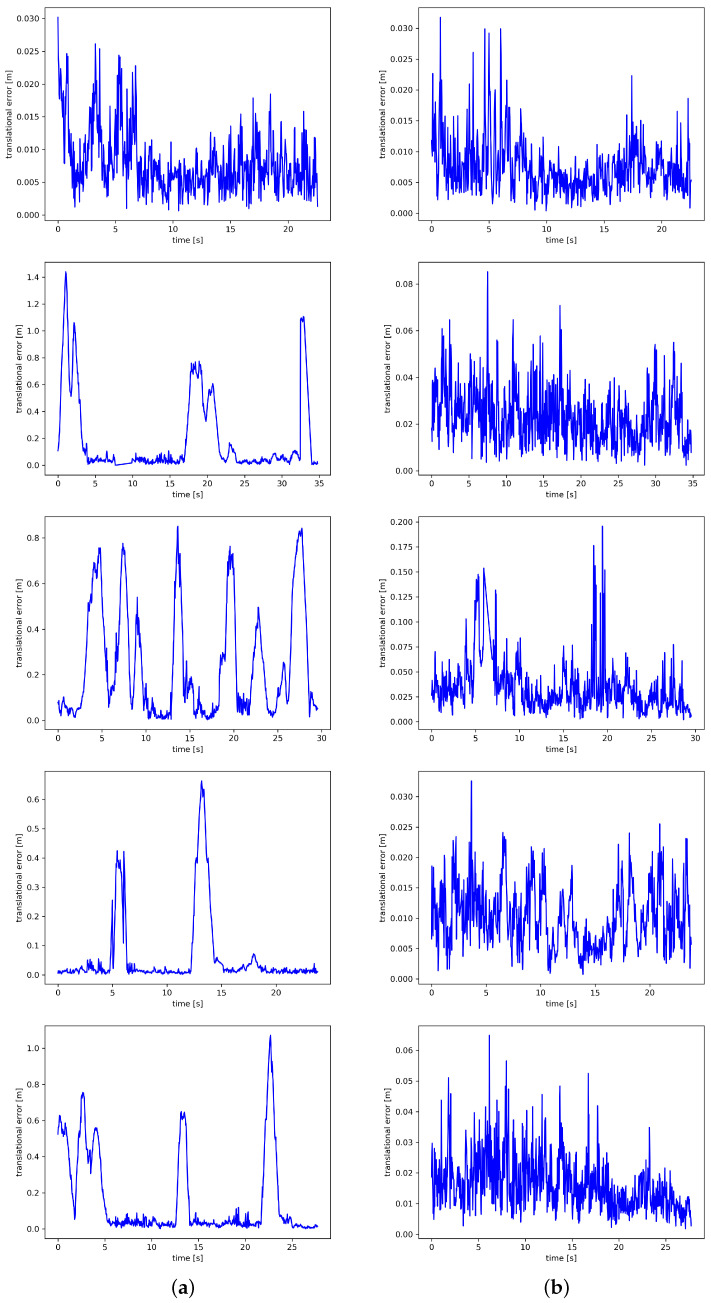
RPE for ORB-SLAM3 and BY-SLAM is evaluated on certain sequences from the TUM RGB-D dataset. In the RPE evaluation, the blue lines denote the RPE at each moment. Different scenes are represented from top to bottom: (1) fr3_sitting_static, (2) fr3_walking_half, (3) fr3_walking_rpy, (4) fr3_walking_static, and (5) fr3_walking_xyz. (**a**) ORB-SLAM3 algorithm results; (**b**) BY-SLAM algorithm results.

Figure 10 and Figure 11 qualitatively depict the estimated and true trajectories using the EVO evaluation tool, showing the ATE and RPE visualization results obtained by ORB-SLAM3 and BY-SLAM, respectively. In Figure 10, it can be directly observed from the figure that the camera trajectory estimated by BY-SLAM is more consistent with the ground truth and better fits the real trajectory. In Figure 11, it can be seen that BY-SLAM is more stable under high dynamic sequences, with a smaller error fluctuation range, and is smoother and more uniform compared to ORB-SLAM3. In summary, the qualitative and quantitative results are consistent, and the experimental results show that BY-SLAM exhibits better performance than the ORB-SLAM3 algorithm in both low dynamic and high dynamic scenarios.

To further validate the effectiveness of the algorithm, the performance of BY-SLAM is compared with that of DynaSLAM, DS-SLAM, RDS-SLAM, the algorithm from ref. [12], Dynamic-VINS, and DeFlowSLAM. Since the RMSE directly measures the degree of deviation between the estimated value and the true value, the RMSE in the ATE of each SLAM system is used for comparison. The experimental results are shown in Table 8.

From Table 8, it can be concluded that the RMSE values of BY-SLAM in the high dynamic sequences w_half, w_rpy, and w_xyz are smaller than those of DynaSLAM, DS-SLAM, RDS-SLAM, ref. [12], Dynamic-VINS, and DeFlowSLAM. Only in the w_static sequence is the RMSE slightly higher than that of DynaSLAM. In the low dynamic s_static sequence, the RMSE of BY-SLAM is essentially consistent with that of other excellent algorithms, indicating that BY-SLAM has good robustness.

### 4.5. Time Evaluation

Table 9 shows the runtime comparison of BY-SLAM with the ORB-SLAM3, DynaSLAM, DS-SLAM and, RDS-SLAM algorithms on the TUM RGB-D dataset, including semantic/detection time and the time required for tracking each frame. The DynaSLAM, DS-SLAM, and RDS-SLAM algorithms’ runtime data are taken from their original papers. Considering the differences in times when the SLAM systems run different sequences, we provide the slowest and fastest runtimes of BY-SLAM and ORB-SLAM3 for each sequence in Table 9. The detection time of BY-SLAM is 34.2 ms, and the time required for tracking time for each frame ranges from 48.6 to 52.3 ms, which is good, real-time performance. This is due to the fact that the semantic information extraction and tracking threads are processed in parallel in this paper, which allows similar speeds to DS-SLAM to be achieved even when only the CPU is used.

### 4.6. Dense 3D Map Construction Experiment

**Figure 12:**
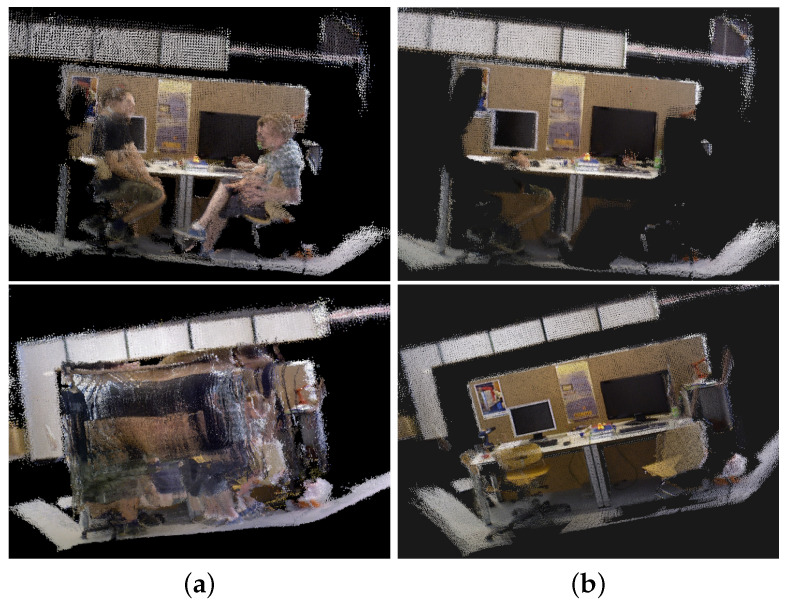
Dense 3D maps for the TUM RGB-D dataset. Different scenes are represented from top to bottom: (1) fr3_sitting_static and (2) fr3_walking_static. (**a**) ORB-SLAM3; (**b**) BY-SLAM.

**Figure 13:**
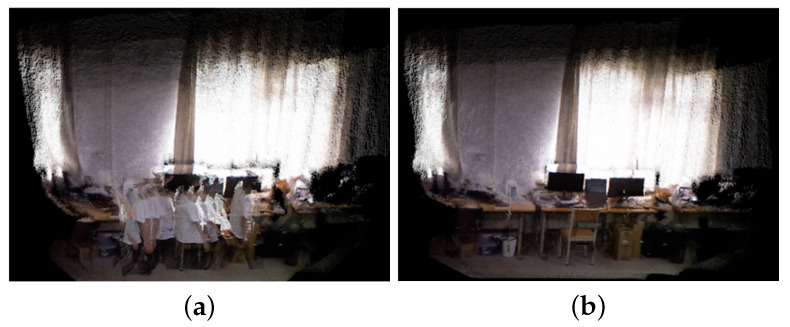
Dense 3D maps of the real-world scene. (**a**) ORB-SLAM3; (**b**) BY-SLAM.

In this section, we test and validate the construction method of dense 3D maps using the TUM RGB-D and real-world scene datasets. The maps are constructed using BY-SLAM and ORB-SLAM3 for comparison. The dense 3D maps constructed from the TUM RGB-D dataset are shown in Figure 12. The dense 3D maps constructed from the real-world scene are shown in Figure 13.

As can be seen from Figure 12 and Figure 13, the ghosting phenomenon caused by human motion in the dense 3D maps constructed by ORB-SLAM3 is quite serious. It is basically impossible to correctly recognize the background information. This is because ORB-SLAM3 does not filter out dynamic objects during map construction, resulting in the retention of a large number of dynamic pixels and the formation of ghosting. In contrast, the dense 3D maps constructed by BY-SLAM have fewer ghosts, less redundancy, and clearer background outlines. This indicates that the dynamic object elimination strategy of BY-SLAM is effective and that the constructed dense 3D maps are more readable.

## 5. Conclusions

In this paper, we propose a visual SLAM system for dynamic environments called BY-SLAM. The system introduces BEBLID instead of the rBRIEF descriptor in the tracking thread of ORB-SLAM3. This method not only improves the matching accuracy of feature points but also reduces the time consumed for feature matching. Additionally, a dynamic object detection thread and a dynamic feature point elimination module are added to the ORB-SLAM3 framework. In this thread, FasterNet is used to replace the backbone network of YOLOv8s, reducing memory access and redundant computation, which improves the inference speed of object detection when using the CPU. The object detection results are then clustered using the DBSCAN algorithm to obtain a finer mask. This approach addresses the over-elimination of static feature points in the object detection frame, thus improving the pose estimation accuracy. After determining the mask of the a priori dynamic target, our model combines the mask with epipolar constraints to accurately eliminate the dynamic feature points within the mask, thereby ensuring that only static feature points are used for localization and tracking. Finally, a new dense 3D map building thread is added to SLAM system; it constructs a 3D dense map with dynamic targets excluded. Evaluated on the TUM RGB-D dataset and real-world scenarios, the results show that our proposed BY-SLAM improves pose estimation accuracy by more than 90% compared to ORB-SLAM3 in highly dynamic scenarios. Additionally, BY-SLAM demonstrates higher robustness and accuracy in pose estimation experiments compared to other classical dynamic SLAM algorithms. However, BY-SLAM, as a visual SLAM algorithm based on feature points, often has difficulty with extracting enough feature points when faced with untextured scenes (e.g., white walls), which leads to a degradation in system performance. To address this problem, future work can consider introducing multi-visual-feature-fusion methods that combine point, line, and surface features for pose estimation. These methods can better adapt to textureless scenes and provide more robust features, thus improving the accuracy and robustness of the system’s pose estimation.

## Figures and Tables

**Table 1:** Performance comparison of different network models on various devices.

Network	Params (M)	FLOPs (G)	Throughput on GPU (fps)	Latency on CPU (ms)	Latency on ARM (ms)	Acc. (%)
MobileViT	1.3	0.42	2393	30.8	348	69.0
MobileNetV2	3.5	0.31	4198	12.2	442	72.0
PVT	13.2	1.94	1266	55.6	708	75.1
FasterNet	3.9	0.34	6807	9.2	143	71.9

**Table 2:** Comparison of feature matching accuracy.

Method	Scene	Matches	Inliers	Rate	Time (s)
ORB	(1)	243	174	71.5%	0.02547
	(2)	204	148	72.6%	0.02526
	(3)	196	121	61.7%	0.02461
ours	(1)	282	231	81.9%	0.01925
	(2)	193	166	86.0%	0.01789
	(3)	196	136	69.4%	0.01833

**Table 3:** Training parameters for YOLOv8s-FasterNet.

Training Parameter	Value
learning rate	0.01
Adam optimizer momentum	0.937
weight decay coefficient	0.0005
batch size	16
epochs	500
box loss	7.5
cls loss	0.5
iou	0.7

**Table 4:** YOLOv8s-FasterNet performance on the CPU.

Model	Size	mAP 50-90	Each Frame (ms)	Params (M)	FLOPs (B)
YOLOv8n	640	37.3	28.9	3.2	8.7
YOLOv8s	640	44.9	64.4	11.2	28.6
YOLOv8m	640	50.2	167.4	25.9	78.9
YOLOv8l	640	52.9	349.4	43.7	165.2
YOLOv8x	640	53.9	460.2	68.2	257.8
YOLOv8s-FasterNet	640	42.1	34.2	6.0	16.3

**Table 5:** Absolute trajectory error (ATE) experimental results.

Sequences	ORB-SLAM3				BY-SLAM				Improvements			
	RMSE	Mean	Median	Std	RMSE	Mean	Median	Std	RMSE	Mean	Median	Std
w_half	0.4474	0.4267	0.4336	0.1846	0.0282	0.0248	0.0220	0.0136	93.70%	94.19%	94.93%	92.63%
w_rpy	0.6809	0.5789	0.5998	0.3585	0.0370	0.0283	0.0215	0.0239	94.57%	95.11%	96.42%	93.33%
w_static	0.3123	0.2779	0.3142	0.1425	0.0101	0.0092	0.0083	0.0042	96.77%	96.69%	97.36%	97.05%
w_xyz	0.4877	0.4126	0.3259	0.2600	0.0143	0.0125	0.0113	0.0070	97.07%	96.97%	96.53%	97.31%
s_static	0.0103	0.0090	0.0079	0.0050	0.0066	0.0058	0.0051	0.0035	33.98%	35.56%	35.44%	30.00%

**Table 6:** Relative translational trajectory error (RPE) experimental results.

Sequences	ORB-SLAM3				BY-SLAM				Improvements			
	RMSE	Mean	Median	Std	RMSE	Mean	Median	Std	RMSE	Mean	Median	Std
w_half	0.4384	0.3652	0.3468	0.2648	0.0354	0.0334	0.0331	0.0117	91.93%	90.85%	90.46%	95.58%
w_rpy	0.4602	0.3596	0.3308	0.2708	0.0632	0.0488	0.0297	0.0402	86.27%	86.43%	91.02%	85.16%
w_static	0.2974	0.2367	0.2091	0.1800	0.0087	0.0078	0.0078	0.0040	97.07%	96.70%	96.27%	97.78%
w_xyz	0.3837	0.2518	0.2351	0.1995	0.0240	0.0229	0.0237	0.0069	93.75%	90.91%	89.92%	96.54%
s_static	0.0143	0.0140	0.0140	0.0037	0.0121	0.0111	0.0111	0.0023	15.38%	20.71%	20.71%	37.84%

**Table 7:** Relative rotational trajectory error (RPE) experimental results.

Sequences	ORB-SLAM3				BY-SLAM				Improvements			
	RMSE	Mean	Median	Std	RMSE	Mean	Median	Std	RMSE	Mean	Median	Std
w_half	7.6875	6.2173	6.2342	6.0702	1.0259	0.9461	0.8641	0.3966	86.65%	84.78%	86.14%	93.47%
w_rpy	7.9519	6.4803	5.8742	4.6086	1.1863	0.9711	0.5779	0.6814	85.08%	85.01%	90.16%	85.21%
w_static	5.0112	3.9581	3.0656	3.0733	0.1173	0.1172	0.1172	0.0056	97.66%	97.04%	96.18%	99.82%
w_xyz	7.1282	4.6363	4.8219	5.4144	0.5940	0.5169	0.4017	0.2927	91.67%	88.85%	91.67%	94.59%
s_static	0.3178	0.3128	0.3128	0.0560	0.2703	0.2445	0.2445	0.0352	14.95%	21.84%	21.84%	37.14%

**Table 8:** Comparison of ATE of each improved SLAM system.

Sequences	DynaSLAM [20]	DS-SLAM [21]	RDS-SLAM [18]	Ref. [12]	Dynamic-VINS [25]	DeFlowSLAM [26]	BY-SLAM
w_half	0.0302	0.0303	0.0807	0.3116	0.0608	0.4200	**0.0282**
w_rpy	0.0417	0.4442	0.1604	0.4983	0.0629	0.0570	**0.0370**
w_static	**0.0068**	0.0081	0.0206	0.3080	0.0077	0.0070	0.0101
w_xyz	0.0156	0.0247	0.0213	0.3047	0.0486	0.0180	**0.0143**
s_static	**0.0065**	0.0069	0.0084	0.0666	-	0.0070	0.0066

Note: Bold values indicate the best results for the current sequence.

**Table 9:** Time evaluation.

Method	Platform	Semantic/Detection Time (ms)	Tracking Time for Each Frame (ms)
ORB-SLAM3 [9]	Intel i9 CPU	-	11.3–14.1
DynaSLAM [20]	CPU, Tesla M40 GPU	195	>300
DS-SLAM [21]	Intel i7 CPU, P4000 GPU	37.57	>65
RDS-SLAM [18]	CPU, RTX2080Ti	30	>50
BY-SLAM	Intel i9 CPU	34.2	48.6–52.3

## Data Availability

The TUM RGB-D dataset can be accessed at https://cvg.cit.tum.de/data/datasets/rgbd-dataset (accessed on 19 April 2023); the HPatches dataset can be accessed at https://github.com/hpatches/hpatches-dataset (accessed on 6 October 2023); the COCO dataset can be accessed at: https://cocodataset.org/#download (accessed on 11 August 2023).
